# CT-Based Thymic Morphology as an Imaging Surrogate of Immune Ageing and Its Association with Coronary Artery Calcification—A Hypothesis-Generating Observational Study

**DOI:** 10.3390/biomedicines14040883

**Published:** 2026-04-13

**Authors:** Isabella Luisa Walther, Karim Mostafa, Agreen Horr, Sandra Freitag-Wolf, Hatim Seoudy, Oliver J. Müller, Sarah Krutmann, Olav Jansen, Patrick Langguth

**Affiliations:** 1 Department of Radiology and Neuroradiology, University Hospital Schleswig-Holstein, 24105 Kiel, Germany; isabellaluisa.walther@uksh.de (I.L.W.); agreen.horr@uksh.de (A.H.);; 2 Institute for Medical Informatics and Statistics, Christian Albrechts University of Kiel, 24118 Kiel, Germany; 3 Department of Internal Medicine III, Cardiology and Critical Care, University Hospital Schleswig-Holstein, 24105 Kiel, Germany; 4 German Centre for Cardiovascular Research (DZHK), Partner Site Hamburg/Kiel/Lübeck, 24105 Kiel, Germany; 5 Department of Internal Medicine V, Angiology, University Hospital Schleswig-Holstein, 24105 Kiel, Germany; 6 Department of Neurology, University Hospital Schleswig-Holstein, 24105 Kiel, Germany; 7 Clinician Scientist Program, Faculty of Medicine, Christian Albrechts University of Kiel, 24118 Kiel, Germany

**Keywords:** thymic involution, coronary artery calcification, inflammaging, immunosenescence, computed tomography

## Abstract

**Purpose:** Thymic involution, a hallmark of immune ageing, is associated with chronic low-grade inflammation (“inflammaging”) and has been implicated in age-associated inflammatory diseases, including atherosclerosis. This study aimed to evaluate the association between persistent thymus and coronary artery calcification based on the Agatston score. **Materials and Methods:** In an exploratory effort, we retrospectively analyzed 206 patients aged 40–64 years who underwent ECG-triggered thoracic CT between 2019 and 2024. Coronary artery calcifications were quantified on virtual non-contrast reconstructions using the Agatston score. Thymic tissue was graded on a five-point scale based on the extent of fatty replacement, with higher grades indicating greater thymic preservation. **Results:** The cohort included 126 men and 80 women. Complete fatty replacement of the thymus (Grade 0) was seen more often in men compared to women (51/126 vs. 18/80; *p* = 0.011). Linear regression analysis revealed a significant inverse association between thymus grade and coronary Agatston score (Beta (B) = −28.8 (95% CI −45.3 to −12.3); *p* = 0.001). After adjusting for age and sex, higher thymic grades remained significantly associated with lower coronary Agatston scores (B = −22.2 (95% CI: −41.7 to −2.6); *p* = 0.03). Further analysis with adjustments for cardiovascular risk factors was not performed. **Conclusions:** Residual thymic tissue was significantly inversely associated with coronary artery calcification, and this association persisted after adjustment for age and sex. These findings support the hypothetical concept that morphologically detectable thymic remnants may reflect interindividual differences in immune ageing and inflammaging that are associated with age-related inflammatory disease phenotypes. The results of this hypothesis-generating study give incentive to further investigate the nature and strength of these associations in prospective studies.

## 1. Introduction

Ageing of the immune system is increasingly recognized as a central driver of organismal ageing and age-associated disease. A hallmark of immune ageing is thymic involution, the progressive age-related regression of the thymus, which leads to a decline in output of naïve T cells and a contraction of the adaptive immune repertoire [1]. This process contributes to immunosenescence and the development of a chronic, low-grade inflammatory state commonly referred to as inflammaging, which has been implicated in a wide range of age-related pathologies, including cardiovascular conditions and atherosclerosis [2,3].

Furthermore, the thymus plays a crucial role in the development and function of regulatory T cells (Tregs), which are essential for maintaining immune tolerance and suppressing inflammatory responses [1,4]. Studies have demonstrated that Tregs exert beneficial effects in cardiovascular disease, including atherosclerosis, by rapidly responding to inflammatory signals, suppressing immune activation, and facilitating the repair of damaged tissues [4,5]. Reduced thymic function leads to a decline in the Treg cell population, which may reduce its atheroprotective capacity which in turn potentially contributes to disease progression [1,6].

Age-associated diseases are increasingly understood to have a substantial immune and inflammatory component [7]. Chronic vascular inflammation, driven in part by immune ageing and dysregulated immune responses, plays a central role in the initiation and progression of atherosclerotic disease. In this framework, coronary artery calcification represents the cumulative structural manifestation of long-standing vascular inflammation and has been proposed as an imaging surrogate of chronic inflammatory burden in ageing populations [7,8,9]. While traditional risk factors such as hypertension and dyslipidaemia are well-established, emerging evidence suggests that age-related alteration of thymic function plays a pivotal role in modulating immune responses that influence the progression of atherosclerosis [9,10].

Thymic morphology can be assessed non-invasively using computed tomography (CT), where age-related fatty degeneration of thymic tissue can be graded using established scoring systems [11,12,13]. CT-based assessment of residual thymic tissue may thus provide a structural imaging surrogate of immune ageing in vivo.

Against this background, the present hypothesis-generating observational study aimed to investigate whether CT-based thymic morphology, as an imaging surrogate of immune ageing, is associated with coronary artery calcification quantified by the Agatston score in middle-aged adults.

## 2. Materials and Methods

### 2.1. Patient Selection

In this study, we retrospectively assessed CT imaging studies of the thorax acquired between 2019 and 2024 in patients aged between 40 and 64 years who had undergone electrocardiogram (ECG)-triggered CT imaging for various clinical indications (e.g., exclusion of aortic dissection or coronary heart disease, planning CT prior to heart valve evaluation, etc.). The prerequisite was that the entire mediastinum was imaged. The age range was set to include a population with an increased likelihood of coronary heart disease (>40 years) with the possible presence of residual thymic tissue. All examinations had to be performed on a Spectral CT system (IQon or 7500, Philips Healthcare, Best, The Netherlands). Patients with pathological alterations in the mediastinum, such as hematomas or tumours, patients after thoracic surgery involving sternotomy, patients with vascular or non-vascular metal foreign bodies in the heart or mediastinum, as well as patients with non-evaluable images, e.g., due severe motion artefacts, were excluded from the analysis.

### 2.2. CT Imaging Protocol

ECG-triggered CT scans were performed on all included patients using spectral CT technology with generation of a spectral-based imaging (SBI) dataset. The following CT standard settings were used: tube voltage 120 kV, tube current time product automatic adjustment 80–250 mAS, slice collimation 2.0 × 128 × 0.6 mm, and pitch factor 0.16–0.3.

As all included CT examinations were performed with intravenous contrast, virtual non-contrast (VNC) images were generated for all patients based on the spectral-based imaging datasets. The contrast-enhanced acquisition phases varied according to the clinical indication; however, coronary artery calcification was assessed exclusively on the VNC images, rendering the specific contrast phase irrelevant for this analysis. Previous studies have demonstrated that VNC images derived from spectral CT allow reliable quantification of coronary artery calcification, showing high correlation with conventional true non-contrast CT used for coronary calcium scoring [14,15]. The VNC images were subsequently analyzed in a standardized manner to assess coronary artery calcification and calculate the Agatston score.

### 2.3. Image Analysis of Coronary Artery Calcification

The VNC images were imported into the cvi42 cardiac analysis software (Circle Cardiovascular Imaging Inc., Calgary, AB, Canada, Version 5.15). Manual segmentation of all coronary calcifications was performed for each coronary artery. The extent of the calcifications was then quantified using the established Agatston scoring method [14].

### 2.4. Image Grading of the Thymic Tissue

**Figure 1:**
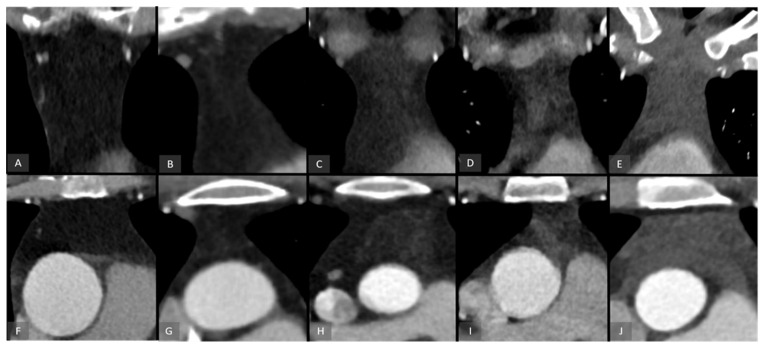
Representative examples of age-related thymic involution. ((**A**,**F**) = 0; (**B**,**G**) = 1, (**C**,**H**) = 2; (**D**,**I**) = 3; (**E**,**J**) = 4). 3D-reconstructed images allow for the visualization of the entire thymic region in the coronal (**A**–**E**) and axial (**F**–**J**) view.

Thymic tissue in the anterior mediastinum was assessed by three radiologists (P.L., I.L.W. and K.M.; up to 13 years of experience) in consensus reading and graded on a five-point scale from 0 to 4 according to the proportion of remaining thymic tissue (Figure 1).

- Grade 0: Complete fatty replacement of the thymus;
- Grade 1: Predominantly fatty tissue with minimal residual thymic components (<25%);
- Grade 2: Mixture of residual thymic tissue and fatty tissue components (25–50%);
- Grade 3: Residual thymic tissue with minimal fatty tissue components (50–75%);
- Grade 4: Almost complete residual thymic tissue (>75%).

In this study, we opted for a five-point scale to improve accuracy in the assessment of thymic involution. Data on interobserver variability was not gathered, since the reading of the thoracic imaging studies was performed as consensus reading. Further assessment of the described five-point-scale was not performed in the framework of this study.

### 2.5. Statistical Analysis

Data management of our cohort was done with Microsoft Excel. Descriptive statistical analysis of our cohort was conducted using IBM SPSS 24. Descriptive data are presented in mean and standard deviation or range. Continuous data were compared with the Mann–Whitney U-Test. Ordinal scaled data were compared using the Chi-Squared or the Fisher Exact Test, as appropriate. Linear regression analysis was performed to assess the association between residual thymic tissue and the presence of coronary calcifications. To assess the impact of patient’s age and sex upon this relation, it was included as a covariate in the linear regression model to test for independence. Interaction effects on the influence of thymic tissue on coronary artery calcifications were calculated for age and sex. We did not perform further analyses corrected for cardiovascular risk factors, since information on these factors was not available. Interobserver variability was not statistically assessed, since image reading with grading of the thymus was performed as consensus reading for all cases. Statistical significance was defined as a *p*-value < 0.05.

## 3. Results

**Figure 2:**
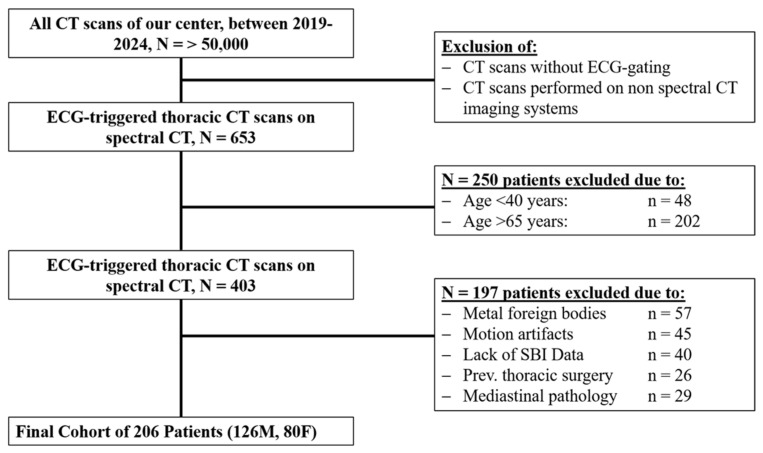
Patient selection flowchart.

**Figure 3:**
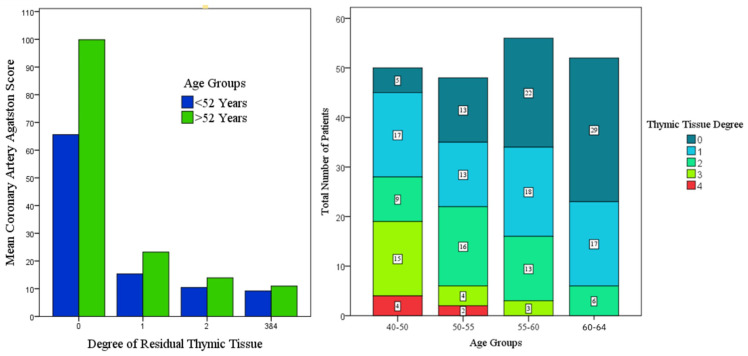
(**Left**): Mean coronary artery Agatston scores by thymic tissue grade, stratified by age group (<52 years and >52 years). Grades 3 and 4 of the residual thymus tissue were combined due to the small number of cases. (**Right**): Distribution of thymic tissue grades across age groups (40–64 years), illustrating the progressive decline in residual thymic tissue with increasing age.

A total of 206 patients were included in the study, compromising 126 men and 80 women (Figure 2). The mean age was 54.8 years (standard deviation 6.8 years). The distribution of thymus grades among the participants was as follows: Grade 0: 69 patients (33.5%); Grade 1: 65 patients (31.6%); Grade 2: 44 patients (21.4%); Grade 3: 22 patients (10.7%); Grade 4: 6 patients (2.9%). A detailed subanalysis of the cohort is shown in Figure 3, Table 1 and in the Supplementary Material in Table S1.

### 3.1. Linear Regression Analysis

**Figure 4:**
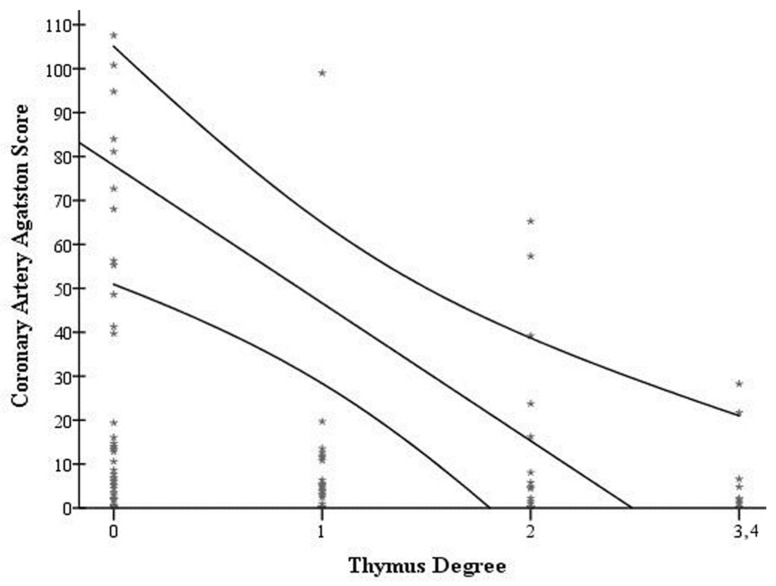
Visual representation of linear regression and 95% CI for assessment of the association of residual thymus tissue on presence of coronary artery calcifications measured with the Agatston score based on the age- and sex-adjusted model.

Linear regression analysis was performed for assessment of the influence of residual thymus tissue on presence of coronary artery calcification (Table 2, Figure 4). In the unadjusted model, a significant inverse association between higher thymic tissue grades and coronary artery calcification was found (regression coefficient Beta (B) = −28.8 (95% CI −45.3 to −12.3; *p* = 0.001). In the next step, the analysis was subsequently adjusted for patient age and sex. When adjusted for age, the association remained statistically significant (B = −25.2 (95% CI −44.5 to −6.0; *p* = 0.01). After further adjustment for patient sex, the association remained statistically significant (B = −22.2 (95% CI: −41.7 to −2.6); *p* = 0.03; Table 2, Figure 4).

In the final step, we performed further analysis on interaction effects of age and sex on the inverse association of thymus degree and coronary calcification, whereby neither age nor sex showed a significant interaction. As previously mentioned, we did not perform further multivariable analysis to assess the potential influence of cardiovascular risk factors.

### 3.2. Kruskal–Wallis Pairwise Comparison

Agatston scores differed significantly across thymus grades (Kruskal–Wallis test, *p* < 0.001). Post hoc pairwise comparisons with Bonferroni correction demonstrated significantly higher Agatston scores in patients with complete thymic involution (Grade 0) compared to all other thymus grades (adjusted *p* < 0.001 for all comparisons; Supplementary Table S2). No significant differences were observed between the remaining thymus grades. Thymus grades 3 and 4 have been combined for this analysis as shown in Figure 3.

## 4. Discussion

This study found a significant inverse association between persistent thymus and the extent of coronary artery calcifications, which remained significant after adjustment for age and sex. Although the absence of further testing including clinical data and cardiovascular risk factors, these results give rise to the notion that age-related thymic involution, as assessed on CT images, may be associated with structural manifestations of chronic vascular inflammation. CT-based thymic morphology may therefore serve as a non-invasive structural surrogate reflecting aspects of immune ageing. This inverse association may reflect the immunological role of residual thymic tissue in shaping immune homeostasis, particularly through the sustained output of Tregs, which have been implicated in the modulation of chronic inflammatory processes [1,3,4]. These observations are consistent with established concepts of inflammaging and immunosenescence, which are increasingly recognized as contributors to age-associated disease, including atherosclerosis [2,3]. Nevertheless, the present study does not allow causal inference, and shared underlying factors may influence both thymic involution and coronary calcification.

The age-related involution of the thymus is one of the most characteristic features of immune ageing. In humans, this involution process presumably begins in infancy, around the first year of life [15,16,17,18]. Progressive fatty degeneration of the thymus is associated with reduced naïve T-cell output and depletion of the peripheral T-cell repertoire. This results in a functional weakening of the adaptive immune response, which in turn is associated with increased susceptibility to infections, malignancies and autoimmune disorders [16,19,20,21,22].

Experimental and clinical studies have highlighted the persistence of residual thymic activity into adulthood and its potential relevance for immune function [1]. In this context, thymic morphology may reflect interindividual differences in immune ageing trajectories rather than serve as a direct determinant of disease. A deeper understanding of how structural thymic changes and degree of residual thymic tissue relate to immune function may therefore provide insight into mechanisms linking immune ageing with age-associated inflammatory conditions, particularly regarding its influence on the pathogenesis of atherosclerosis.

Tregs play a central role in maintaining immune tolerance and controlling excessive inflammation. Prior work has demonstrated associations between Treg dysfunction, immune imbalance, and atherosclerotic disease [1,23,24]. Novel immune markers such as the neutrophil-to-lymphocyte and platelet-to-lymphocyte ratio have been proposed as indicators of immune dysregulation in cardiovascular diseases [25,26,27,28]. In this framework, the results we obtained in our study extend this concept by suggesting that immune-ageing-related changes can also be traced morphologically, with preserved thymic tissue on CT being associated with lower degrees of coronary artery calcification. This observation underscores the potential relevance of thymic morphology as a structural correlate of immune ageing in the context of vascular ageing. However, further testing is needed to confirm this association to be independent of cardiovascular risk factors.

Lifestyle and metabolic factors are known to influence immune ageing. Data from a Swedish cohort of individuals aged between 50 and 64 years demonstrated associations between obesity, dietary factors and accelerated thymic involution [12]. Given the comparable age range of our cohort, the findings of the mentioned study support the notion that thymic morphology may reflect integrated effects of immune and metabolic ageing rather than being a single isolated process. This perspective underscores the importance of considering thymic morphology within a broader biological context, where immune ageing interacts with metabolic and environmental factors. The exploratory results of our study of a potential association between atherosclerosis and residual thymic tissue lay the groundwork for future studies combining CT-based thymic assessment with metabolic parameters—such as body composition, fat distribution and immunological profiling. These further investigations are needed to confirm the hypothesis generated in the presented study, and they may help to further elucidate the mechanisms linking immune ageing with age-associated inflammatory disease.

Several limitations should be acknowledged. First, coronary artery calcification was assessed using VNC images, which may underestimate coronary artery calcification when compared to true non-contrast images [29]. While this represents a technical limitation, prior studies have shown good correlation between VNC- and true non-contrast-derived Agatston scores, supporting its validity for population-level analysis [29,30,31]. Second, the absence of detailed lifestyle and cardiovascular risk factor data limited adjustment for potential confounders. Third, statistical analysis was limited to linear regression analysis with interaction effect testing. Given the lack of information on clinical risk factors, we did not perform multivariate regression analysis to further explore the influence of clinical and cardiovascular factors. Fourth, imaging assessment was performed by consensus reading, as this study did not primarily aim at establishing or evaluating the performance of the previously published similar scales for grading of residual thymic tissue [32]. Fifth, functional immune parameters were not available, precluding direct assessment of thymic output or immune competence. Finally, non-calcified coronary artery plaques were not evaluated, limiting the assessment of the full atherosclerotic burden.

This hypothesis-generating study suggests that greater preservation of thymic tissue on CT is associated with a lower burden of coronary artery calcification, an established structural marker of vascular ageing. These findings support the concept that immune and vascular ageing are interrelated processes and suggest that thymic morphology may reflect interindividual differences in age-associated inflammatory burden; however, a causal relationship cannot be proven with our data. While the present data do not allow conclusions regarding causality or clinical prognosis, they highlight CT-based thymic assessment as a potential imaging correlate linking immune ageing with structural manifestations of cardiovascular ageing. Importantly, the unavailability of clinical patient data has to be mentioned as the main limiting factor of this study.

Future studies should include larger prospective cohorts and integrated imaging-based thymic assessment with functional immunological measurements and detailed clinical phenotyping. Combining imaging-derived thymic morphology with immune profiling, metabolic parameters, and longitudinal data may help to further clarify the relationship between immune ageing and structural manifestations of vascular ageing. In this context, incorporation of additional cardiovascular and lifestyle-related factors, as well as evaluation of non-calcified coronary plaque burden, is necessary in order to achieve a more complete understanding of the mechanisms linking thymic involution with age-associated inflammatory disease and to clarify the role of potential clinical confounding factors.

## Figures and Tables

**Table 1:** Descriptive overview of the cohort. * indicates significant results. The Agatston score is presented both as median and interquartile range as well as mean and range for completeness.

	Overall (n = 206)	Male (n = 126)	Female (n = 80)	*p*
Age	54.6 ± 6.8	54.5 ± 6.5	54.7 ± 7.2	0.694
Mean Agatston Score	41.9 (0–963.9)	57.1 (0–963.2)	18.1 (0–895.8)	n.a.
Median Agatstson Score	0, IQR 11.6	0.4 IQR 31.1	0, IQR 3.1	0.007 *
Thymus Grade 0	69 (33.5%)	51 (40.5%)	18 (22.5%)	0.011 *
Thymus Grade 1	65 (31.5%)	35 (27.8%)	30 (37.5%)	0.162
Thymus Grade 2	44 (21.4%)	26 (20.6%)	18 (22.5%)	0.758
Thymus Grade 3	22 (10.7%)	12 (9.5%)	10 (12.5%)	0.502
Thymus Grade 4	6 (3.9%)	2 (1.6%)	4 (5.0%)	0.207

**Table 2:** Linear regression analysis for influence of thymus degrees on coronary artery calcification. * indicates significant results.

Adjustment	Beta	*p*	95% CI Lower	95% CI Upper
Unadjusted	−28.8	0.001 *	−45.3	−12.3
Adjusted for Age	−25.2	0.01 *	−44.5	−6.0
Adjusted for Age and Sex	−22.2	0.03 *	−41.7	−2.6
**Interaction Effects**				
Age Interaction Effect	−0.7	0.402	−2.4	0.95
Sex Interaction Effect	5.8	0.614	−16.8	28.3

## Data Availability

The datasets used and analyzed during the current study are available from the corresponding author on request.

## Supplementary Materials

The following supporting information can be downloaded at: https://www.mdpi.com/article/10.3390/biomedicines14040883/s1, Table S1: Patient sex distribution, age, and coronary calcium score stratified by thymus grade. Age is given as mean ± SD. Agatston score is presented as mean (range); Table S2: Kruskal–Wallis pairwise comparisons. Thymus grades 3 and 4 were combined due to small sample size. Pairwise comparisons were performed using Dunn–Bonferroni correction following a significant Kruskal–Wallis test.

## Abbreviations

The following abbreviations are used in this manuscript:

CI	Confidence interval
CT	Computed tomography
ECG	Electrocardiogram
SBI	Spectral-based imaging
Tregs	Regulatory T cells
VNC	Virtual non-contrast
