# Supplementary material for: CT-Based Thymic Morphology as an Imaging Surrogate of Immune Ageing and Its Association with Coronary Artery Calcification—A Hypothesis-Generating Observational Study
Source: Biomedicines. 2026 Apr 13;14(4):883. doi: 10.3390/biomedicines14040883 (PMC13113726; doi:10.3390/biomedicines14040883)
Supplement: Supplementary file 1 [file biomedicines-14-00883-s001.zip › biomedicines-4190097-supplementary.pdf]

Variable	Thymus 0 (n=69)	Thymus 1 (n=65)	Thymus 2 (n=44)	Thymus 3 (n=22)	Thymus 4 (n=6)
**Sex, n (%)**					
Male	51 (73.9%)	35 (53.8%)	26 (59.1%)	12 (54.5%)	2 (33.3%)
Female	18 (26.1%)	30 (46.2%)	18 (40.9%)	10 (45.5%)	4 (66.7%)
**Age (years)**	58.2 ± 5.0	54.6 ± 6.5	54.0 ± 5.5	46.9 ± 6.2	45.1 ± 6.0
**Agatston Score**	94.9 (0 – 963.9)	20.6 (0 – 311.1)	12.7 (0 – 170.4)	8.5 (0 – 123.4)	0.8 (0 – 4.8)

Supplementary Table S1. Patient sex distribution, age, and coronary calcium score stratified by thymus grade. Age is given as mean ± SD. Agatston score is presented as mean (range).

Group Comparison	Test Statistic	Std. Error	Standardized Test Statistic	p-value	Adjusted p-value (Bonferroni)
**3 vs 0**	49.127	12.328	3.985	<0.001	<0.001*
**2 vs 0**	45.747	10.615	4.310	<0.001	<0.001*
**1 vs 0**	40.237	9.510	4.231	<0.001	<0.001*
3 vs 2	3.380	13.301	0.254	0.799	1.000
3 vs 1	8.890	12.437	0.715	0.475	1.000
2 vs 1	5.510	10.741	0.513	0.608	1.000

Supplementary Table S2. Kruskal–Wallis pairwise comparisons. Thymus grades 3 and 4 were combined due to small sample size. Pairwise comparisons were performed using Dunn–Bonferroni correction following a significant Kruskal–Wallis test.
